# Mid- to Long-Term Outcomes of Arthroscopic Shoulder Stabilization in Athletes: A Systematic Review

**DOI:** 10.3390/jcm12175730

**Published:** 2023-09-02

**Authors:** Muzammil Akhtar, Jimmy Wen, Daniel Razick, Mouhamad Shehabat, Ali Saeed, Osamah Baig, Maaz Asim, Ilham Tokhi, Sonia Aamer, Muhammad Bilal Akhtar

**Affiliations:** 1 College of Medicine, California Northstate University, Elk Grove, CA 95757, USA; jimmy.wen10016@cnsu.edu (J.W.); daniel.razick10009@cnsu.edu (D.R.); mouhamad.shehabat8858@cnsu.edu (M.S.); maazasim125@gmail.com (M.A.); ilhamtokhi@gmail.com (I.T.); 2 College of Osteopathic Medicine, William Carey University, Hattiesburg, MS 39401, USA; asaeed@ucdavis.edu; 3 Lake Erie College of Osteopathic Medicine, Erie, PA 16509, USA; obaig@ucdavis.edu; 4 Southern California Orthopedic Institute, Bakersfield, CA 93309, USA; saaiqbal@ucdavis.edu; 5 Department of Occupational Therapy, University of St. Augustine for Health Sciences, San Marcos, CA 92069, USA; bilalak978@gmail.com

**Keywords:** shoulder arthroscopy, Bankart lesion, athlete, return to sports

## Abstract

There exists a considerable amount of evidence regarding short-term outcomes of shoulder arthroscopy in athletes; however, mid- to long-term data are limited. Therefore, the purpose of this review is to evaluate studies assessing mid- to long-term outcomes and rates of return to sport in athletes undergoing primary shoulder arthroscopy. A search for the systematic review was performed in PubMed, Scopus, and Embase on 14 March 2023. Study parameters, as well as their respective outcomes, were described in detail and compiled into diagrams. Five studies were included, which contained data on a total of 307 shoulders in patients with mean ages ranging from 20.3 to 26.9 years and mean follow-up times ranging from 6.3 to 14 years. The arthroscopic Bankart repair was the primary surgical intervention performed in all five studies. The overall rate of return to sport was 84% (range, 70–100%) across the studies. The rate of return to sport at pre-injury level was 65.2% (range, 40–82.6%) across four studies. The overall rate of recurrent instability was 17.3%, with redislocation specifically occurring in 13.7% of patients across all studies. The overall rate of revision surgery was 11.1%. Athletes who underwent primary shoulder arthroscopy demonstrated favorable outcomes and a high rate of RTS at a minimum follow-up of 5 years. However, rates of recurrent instability, redislocation, and revision surgery occurred at less than favorable numbers, which emphasizes the importance of proper patient selection when considering candidates for arthroscopic versus open repairs.

## 1. Introduction

Shoulder injuries in athletes typically include rotator cuff tears, superior labral anterior posterior (SLAP) tears, biceps tendinitis, anterior and posterior glenohumeral instability, pectoralis major and minor tears, and acromioclavicular joint dislocations and separations [1,2,3]. A recent systematic review of 15 studies in female gymnasts found shoulder injuries to comprise 4.2–7.5% of all injuries [4], whereas another study found that shoulder injuries comprised nearly a third of all sports-related injuries during a 15-year period at a level one trauma center [5]. Anterior glenohumeral dislocations are a common shoulder injury among athletes and have the risk of progressing to recurrent instability due to labral detachment, termed a Bankart lesion. The gold standard surgical treatment for this has historically been the open Bankart repair; however, arthroscopic techniques have become a more popular option due to reduced post-operative pain, earlier rehabilitation, and an earlier return to sports (RTS) [6,7]. In addition to Bankart lesions, SLAP tears and Hill–Sachs lesions may be present in athletes with anterior glenohumeral dislocations and instability [8,9], which require additional repair.

Short-term results and patient-reported outcome measurements (PROM) following arthroscopic shoulder repairs have been well documented and have shown excellent early stabilization post-operatively in athletes. DeBerardino et al. [10] reported that at a mean follow-up of 37 months (24–60 months) after primary shoulder arthroscopy, athletes reported a mean Rowe score of 92 (30–100), a mean single assessment numeric evaluation patient rating of 95.5% (50–100%), and a mean Short-Form-36 (SF-36) physical function score for stable shoulders of 99 (95–100). Of 48 total shoulders, 43 remained stable at follow-up, with 6 failures (recurrent subluxation or dislocation). Factors associated with the six failures included a history of bilateral shoulder instability, a 2+ sulcus sign, and insufficient capsulolabral tissue at the time of repair. All 43 patients with stable shoulders returned to sports at pre-injury level or higher. Harada et al. [11] reported that at a mean follow-up of 39.7 months after arthroscopic Bankart repair, the 24 competitive overhead athletes reported significant improvements in the range of shoulder motion, the Rowe score, the Japanese Shoulder Society Shoulder Instability Score (JSS-SIS), and the Japanese Shoulder Society Shoulder Sports Score (JSS-SSS) at latest follow-up. In total, 15 of the 24 athletes returned to sports at pre-injury level or higher at a mean of 13.3 months. Gerometta et al. [12] reported that at a mean follow-up of 24.4 ± 7.7 months, of 46 patients, 95.7% of them returned to sports at pre-injury level or higher at a mean of 9.8 ± 5.4 months. However, there remains a paucity of studies analyzing the long-term results of patients undergoing arthroscopic shoulder repairs.

Short-term results of shoulder arthroscopy in athletes have been well documented; however, there is limited evidence regarding mid- to long-term outcome. Therefore, the purpose of this review is to evaluate minimum the 5-year outcomes and rate of RTS after primary shoulder arthroscopy in athletes. We hypothesized that athletes who undergo primary shoulder arthroscopy would report favorable PROMs, clinical benefit, and a high rate of RTS at a minimum 5-year follow-up.

## 2. Materials and Methods

This systematic review followed guidelines established by the Preferred Reporting Items for Systematic Reviews and Meta-analyses (PRISMA). A search was performed in three databases on 14 March 2023: PubMed, Scopus, and Embase. Two authors (M.A. and D.I.R.) identified all articles included in the study. The searches used to perform the systematic review in all three databases are reported in Table 1. We did not set any limits on our search strategies.

A PICO (patient, intervention, comparison, outcome) method was used to determine our search strategy. In our study, the patient population was defined as those participating in sports at any level. The intervention was arthroscopic repair of shoulder injuries or pathologies in this athletic population. Since this was not a comparative study, we did not have a control group or a group with an alternative intervention. The outcomes in this study consisted of PROMs, patient satisfaction, overall RTS, RTS at preinjury level, and rates of recurrent instability and revision surgery. Studies were included if they reported on outcomes of athletes at a minimum five-year follow-up after any primary shoulder arthroscopy surgery. Athletes were defined as patients participating in a sport at any level prior to shoulder arthroscopy. Mid-term was defined as a minimum of five years and long-term was defined as a minimum of ten years. Exclusion criteria included case reports, review articles, cadaveric studies, articles not in English, and studies in which patients underwent prior surgical intervention for shoulder problems. Two reviewers (M.A. and D.I.R.) analyzed all articles included in this study and if they were not unanimous in their decision, then articles underwent further review until a consensus was reached to determine article inclusion. All included articles underwent a rigorous reference search to determine whether additional studies could be added to the systematic review. Additionally, a manual search was performed to find additional studies that may have been missed in our systematic search of the three databases. This review was not registered via PROSPERO, nor was a review protocol prepared.

Two authors (M.A. and D.I.R.) used the Methodologic Index for Nonrandomized Studies (MINORS) criteria [13] to score all articles included in the systematic review based on their study quality and to determine risk of bias. The MINORS items are scored 0 (not reported), 1 (reported but inadequate) or 2 (reported and adequate), with a maximum possible score of 16 for non-comparative studies. Each author scored the article individually before reviewing their scores. Discrepancies in scores were resolved by a re-review of the articles until unanimous consensus was reached. If a MINORS criteria was scored a 1 or 2 for seven or more categories, the study was determined to have a low risk of bias. If a score of 1 or 2 was present for five to six categories, the study was determined to have a moderate risk of bias. Finally, if a score of 1 or 2 was found in less than or equal to four categories, then the study was determined to have a high risk of bias. Additionally, levels of evidence for included articles were determined using criteria mentioned by Hohmann et al. [14].

Study variables included in the systematic review included title, author, publication date, study period, study design, number of patients, number of shoulders, mean follow-up time, mean age, sport type, competition level, indications for surgery, radiographic and intraoperative findings, surgical treatment, RTS, PROMs, and rate of secondary shoulder surgery. All extracted data were compiled for analysis and tables were made to visualize the data using Microsoft Word (Microsoft Office 2011; Microsoft, Redmond, WA, USA).

Descriptive statistics (means, percentages, standard deviations) are reported in this review when applicable and when available. A meta-analysis was intended to be performed to compare preoperative and postoperative PROMS, but unfortunately there were not enough data available from the included studies.

## 3. Results

### 3.1. Results of the Literature Search

**Figure 1:**
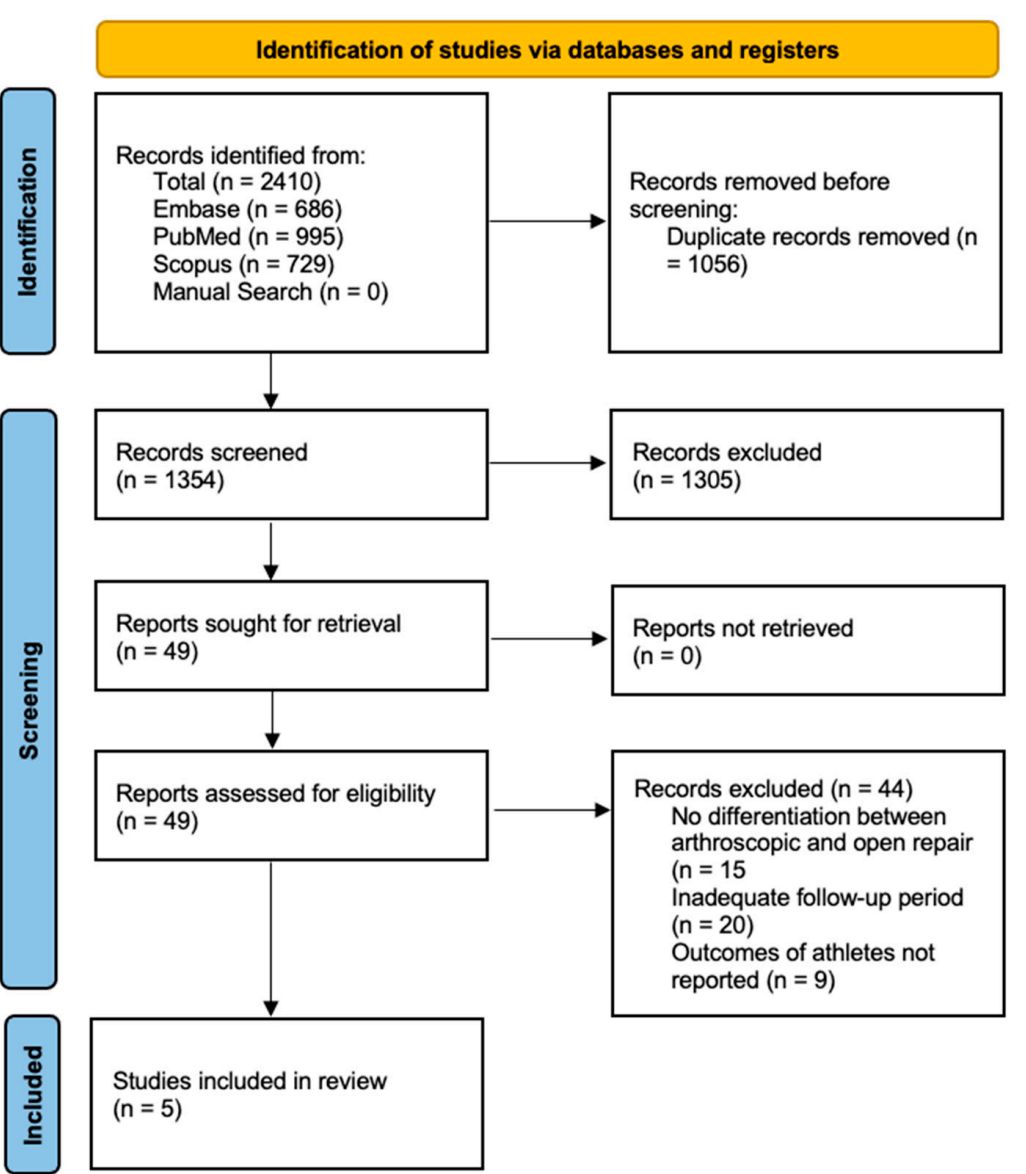
PRISMA flow diagram depicting article selection process.

The initial search revealed 2410 articles through PubMed, Scopus, and EmBase. Next, 1056 duplicates were removed, leaving 1354 articles. A review of the title and abstract of these 1354 articles narrowed our results down to 49 articles. A full-text review of these 49 articles was conducted to determine which ones to include in the systematic review. Five articles met the established inclusion criteria and were included in the study [15,16,17,18,19]. Figure 1 depicts the PRISMA flow diagram for the article search process.

### 3.2. Methodological Quality and Risk of Bias

The MINORS scores for all included studies are summarized in Table 2. One study had a score of 13 [16], three studies had a score of 10 [15,17,18], and another study had a score of 8 [16]. Additionally, the risk of bias was low in one study [16], moderate in three studies [15,17,18], and high in another study [16].

### 3.3. Demographics and Level of Sports Involvement

Demographic data reported in this review included author, publication year, level of evidence (LOE), study type, study period, number of shoulders, mean follow-up time, mean age at surgery, and sports type and competition level, which are recorded in Table 3. One study had an LOE of III [16], while the remaining studies all had an LOE of IV [15,17,18,19]. The study periods among the included studies ranged from as early as 1992 to as late as 2015. The number of shoulders in the included studies ranged from 20 to 144. Follow-up times ranged from 60 to 216 months. The mean or median age at surgery was cited in four studies [15,16,17,18], with the fifth study reporting mean age at time of initial injury [19]. Three studies reported on specific preoperative sport type or competition level [16,17,19], whereas one study reported that the athletes were either in or retired from the military, with one being a collegiate-level soccer player [18].

### 3.4. Intraoperative and Radiographic Findings, and Surgical Outcomes

Surgical indication, surgical intervention, intraoperative findings, PROMs at latest follow-up, radiographic findings at latest follow-up, and patient satisfaction are recorded in Table 4. Indications for shoulder arthroscopy were cited as anterior shoulder instability or dislocation in all five studies, with three studies citing the cause to be traumatic [15,16,18]. The Bankart repair was the primary arthroscopic procedure performed in all five studies, with one study having concomitant radiofrequency capsulorrhaphy due to capsular laxity carried out in two patients [19]. Intraoperative findings were reported in four studies [15,16,17,19], among which there were Bankart lesions, Hill–Sachs lesions, superior labral anterior posterior (SLAP) lesions, labral lesions, Buford complex, and glenoid chondromalacia. All five studies [15,16,17,18,19] reported postoperative PROMs. The most commonly reported PROMs were the Rowe score and the Western Ontario Shoulder Instability Index (WOSI), both of which were reported in three studies. Other reported PROMs included the Constant score, the Visual Analog Scale (VAS), Shoulder-Instability Return to Sport after Injury (SIRSI), the Subjective Shoulder Score (SSV), Single Assessment Numeric Evaluation (SANE), the American Shoulder and Elbow Surgeons (ASES) score, the Simple Shoulder Test (SST), the SF-36 survey, and Disabilities of the Arm, Shoulder, and Hand (DASH). Radiographic findings at the latest follow-up were included in three studies [15,16,19], among which were the visibility of anchors, bone marrow edema, joint effusion, osteoarthritis, glenoid bone loss, degree of arthrosis, visibility of drill holes, and the presence of Hill–Sachs lesions. Three studies reported on the satisfaction rates amongst patients undergoing the arthroscopic procedure, or whether patients would decide to undergo the procedure again [15,16,18].

### 3.5. Factors Associated with Surgical Outcome or Satisfaction

Bauer et al. [15] reported that the Rowe score initially increased to a high of 93.5 at a two-year follow-up, but then proceeded to drop to as low as 83.4 at the latest 14-year follow-up. This fall in the Rowe score is explained by evidence that patients tend to be more careful when using their shoulder up until around two years post-operation [20]. In this cohort, most redislocations tended to occur after the two-year mark, as well, which explains the fall in the Rowe score.

A linear regression by Hurley et al. [16] revealed that the SIRSI score, SSV score, VAS score, and no sleep trouble (*p* < 0.0001, *p* < 0.0001, *p* < 0.0031, *p* = 0.0029, respectively) were associated with satisfaction. Additionally, logistic regression revealed revision surgery, not being able to RTS at pre-injury levels, and redislocation (*p* = 0.0029, *p* = 0.0005, *p* = 0.0031, respectively) were associated with lower satisfaction. Linear regression showed that the SIRSI score, VAS score, and no sleep trouble (*p* < 0.0001, *p* < 0.0001, *p* < 0.0001, respectively) were associated with the SSV score, which measures shoulder function. Additionally, logistic regression determined that RTS at pre-injury levels was associated with SSV score, as well. Overall, the SIRSI score, VAS score, sleep trouble, and ability to RTS were associated with both satisfaction and function (SSV score). Revision surgery and redislocation were, however, only associated with satisfaction and not function. The low VAS score of 2.1 and high SSV score of 85.8 in this cohort indicated that the shoulder operated on felt similar to an uninjured shoulder at the time of follow-up.

Alentorn-Geli et al. [17] reported that regarding the median Rowe score of 80 (25–100), 71% of patients had an excellent result, 19% had a good result, 5% had a fair result, and 5% had a poor result. The six patients with a fair or poor Rowe score were among those who had evidence of redislocations any time after the initial arthroscopic procedure.

Owens et al. [18] reported that for this same cohort in an earlier study at a mean follow-up period of 37 months, the mean SANE score was 95.5 [10], compared to the current study at mean follow-up of 141 months, in which the mean SANE score was 91.7. Analysis via a Student’s t-test of the SANE score at both follow-up periods revealed no significant statistical differences (*p* = 0.10).

Privitera et al. [19] had a comparative design in which the surgical shoulder (SS) was compared with the contralateral healthy control shoulder (CS) in 15 of the 20 patients. The remaining 5 patients had abnormalities in the contralateral shoulder, preventing a comparison between the SS and CS. The total WOSI Score was significantly lower in the SS group compared to the CS group (83% vs. 97%, *p* = 0.008), with similar significant differences found in all WOSI Physical, Sports/recreation, Lifestyle, and Emotions domains (*p* < 0.05). Failed repairs (*n* = 7), defined as those with a postoperative dislocation, revision surgery, or a positive apprehension and relocation sign on examination, had a significantly lower total WOSI score compared to successful repairs (*n* = 13) (55% vs. 92%, *p* = 0.005). Successful repairs (*n* = 13) had significantly lower WOSI Sports/physical scores compared to their CS (*p* = 0.035), whereas differences in all other WOSI domains were insignificant (*p* > 0.05). The three categories of the DASH score which measures disability are the Main, Work, and Sports/arts modules. Greater disability was noted in the Main and Sports/arts module in the SS compared to the CS (*p* < 0.05), whereas a significant difference in disability was not noted in the Work module.

### 3.6. Factors Associated with Radiographic Outcomes at Follow-Up

Bauer et al. [15] reported that though nine patients were found to have developed OA at follow-up, it was considered mild in eight patients, using the Samilson classification. The low overall signs of OA at 28.1% can be explained by the younger cohort, at an average age of 21.6 in this study. It was determined that a younger age at the time of first dislocation, contact, collision, and overhead sports, the size of the Bankart lesion, and visible anchors at the final follow-up were significant risk factors for osteoarthritis (OA) (*p* = 0.007, *p* = 0.007, *p* = 0.049, *p* = 0.033, respectively). Redislocations after the initial arthroscopic surgery occurred in 44.4% of patients with signs of OA (*p* = 0.039). Patients with OA also had significantly lower Constant and Rowe scores (*p* = 0.037, *p* = 0.043, respectively) compared to those without OA. Interestingly, it was found that the rates of OA development occurred more frequently in patients that suffered atraumatic redislocations rather than traumatic redislocations (*p* = 0.008), a finding not previously reported in the literature.

Privitera et al. [19] reported that higher grades of arthrosis were present in the SS vs. the CS (*p* = 0.002). However, no difference was noted between successful (*n* = 13) and failed repairs (*n* = 7), defined as those requiring revision surgery (*p* = 0.167).

### 3.7. Rates of Return to Sport and Recurrent Instability

**Figure 2:**
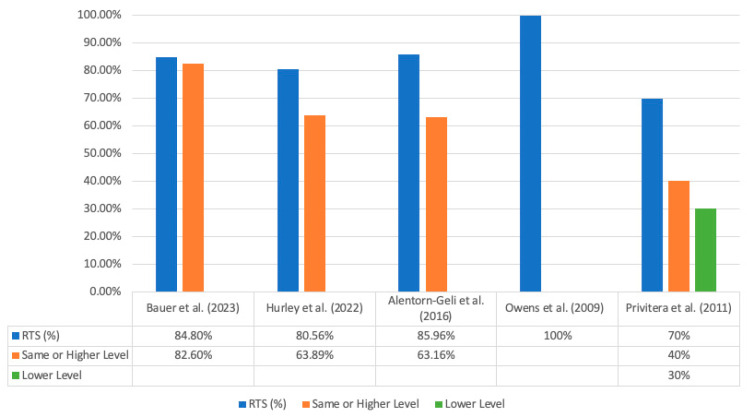
Rates of RTS and levels of performance at follow-up [15,16,17,18,19].

**Figure 3:**
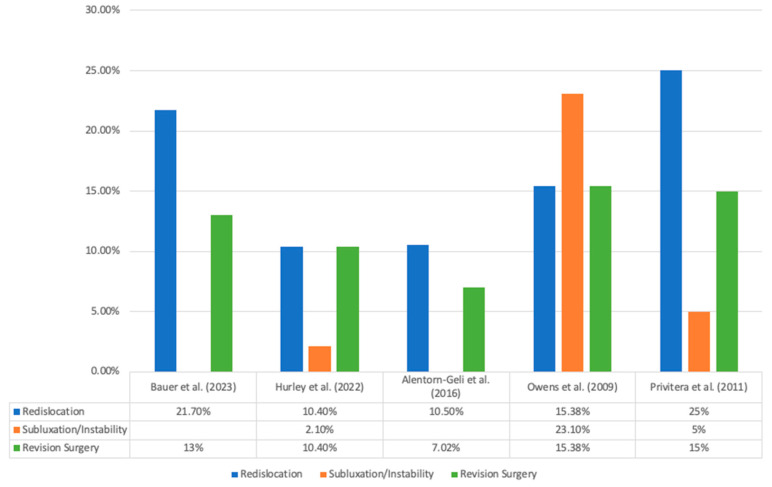
Rates of recurrent instability and revision surgery [15,16,17,18,19].

Rate of RTS, mean time to RTS, rate of recurrent instability, and time from the initial arthroscopic procedure to incidence of recurrent instability are recorded in Table 5. All five studies [15,16,17,18,19] directly reported on the rate of RTS, with the rate of RTS being 84% (257/306) across the studies. Four studies reported on the ability of patients able to return to pre-injury shoulder function [15,16,17,19], with a rate of 65.2% (174/267) across the four studies. The overall rate of recurrent instability was 17.3% (53/306), with redislocation specifically occurring in 13.7% (42/306) of patients across all five studies. The overall rate of revision surgery was 11.1% (34/306). Three studies [15,18,19] reported the mean time from the initial arthroscopic procedure to the event of recurrent instability (redislocation, subluxation, instability), with mean times ranging from 28 months to 50 months. Data regarding RTS and the level of performance at follow-up are summarized in Figure 2. Data regarding rates of recurrent instability and revision surgery are summarized in Figure 3.

### 3.8. Return to Sport and Activity Level at Follow-Up

Bauer et al. [15] measured the level of involvement in sports at the latest follow-up using the sports activity level score established by Valderrabano et al. [21] using the following scale: grade 0, none; grade 1, moderate; grade 2, normal; grade 3, high; and grade 4, elite. The mean Valderrabano score in this cohort of 46 patients at latest follow-up was 1.6 ± 1.1 (1–5 h of sports activity per week). This was a relatively low sport level, which favored a high rate of RTS at 85% in this cohort. Among the seven patients who did not RTS, the sports that were given up included the following: handball (4), American football (1), soccer (1), and wrestling (1).

Alentorn-Geli et al. [17] reported that of the eight patients who did not return to soccer after the arthroscopic procedure, none of their reasons to quit were due to their shoulder. The main reasons to quit were knee injuries (2), changes in personal life (3), and job-related reasons (3).

Owens et al. [18] measured shoulder function at follow-up through performance on the Army Physical Fitness Test (APFT), a three-event test consisting of two minutes of push-ups, two minutes of sit-ups, and a two-mile run, with a maximum total score of 300. The mean APFT at latest follow-up in this study was 287.45, an excellent score. The most amount of push-ups able to be performed in two minutes before the injury was a mean of 77.7 (30–115) at pre-injury and at latest follow-up was a mean of 72.8 (20–100). Additionally, this study assessed the percentage of pre-injury shoulder function among patients, with a reported mean of 93.3% (40–105%). The mean Tegner score at follow-up in this group was 6.5 (3–10). At the time of the latest follow-up, 20 patients remained on active military duty, 18 patients left military service, and 1 patient was a civilian dependent at the time of his surgery who went on to have full shoulder function and play collegiate-level soccer.

Privitera et al. [19] reported that of the six players who did not RTS, four of them quit due to shoulder problems and two quit because they were not interested in their sport any longer, without any complaint regarding their shoulder.

### 3.9. Redislocations and Revision Surgeries

Alentorn-Geli et al. [17] reported that the redislocation rate was higher in patients under 20 years old, at 15.2%, compared to patients older than 20 years, with a redislocation rate of 7.1%. Aside from the studied cohort of 57 patients, 22 patients who could not be followed-up with had no evidence of redislocation up until the last time they were seen in the clinic.

Hurley et al. [16] reported that in addition to revision surgery, the following procedures were also carried out: arthroscopic release (2), arthroscopic rotator cuff repair (2), biceps tenodesis (1), plate fixation for clavicle fracture (1), and subacromial decompression (1).

Owens et al. [18] reported that among the six episodes of recurrent dislocations, one episode occurred in four patients, two episodes occurred in one patient, and three episodes occurred in one patient. Among the nine episodes of recurrent subluxations, one episode occurred in one patient, two episodes occurred in three patients, three episodes occurred in three patients, six episodes occurred in one patient, and twenty episodes occurred in one patient. Among all fifteen patients who experienced episodes of recurrent instability, two were lost to follow-up, and among the remaining thirteen patients, all episodes of recurrent instability occurred during significant athletic activity. Activities associated with recurrent dislocations included volleyball, football (2), water skiing, and military training. Activities associated with subluxation events included tackle football, soccer (2), wrestling (2), softball, basketball, and military obstacle courses. Additionally, among the six patients who underwent revision surgery, four were due to redislocation events and two were due to subluxation events. Among the six patients who underwent revision surgeries, four patients underwent a single open repair, one patient underwent two open repairs, and another patient underwent an arthroscopic repair.

Privitera et al. [19] reported that recurrent postoperative instability was experienced due to trauma to the shoulder in six of seven patients. Additionally, among the three patients who underwent revision surgery in their cohort, two required it due to redislocation and one required it due to frequent subluxation events.

## 4. Discussion

The main findings of this systematic review were that athletes exhibited desirable outcomes following primary shoulder arthroscopy at a minimum follow-up of 5 years, the athletes exhibited a high rate of RTS along with a high rate of RTS at pre-injury level, and incidences of recurrent instability, redislocation, and revision surgery occurred in less-than-desirable numbers.

In this systematic review, there was a high rate of RTS, at 84%, with the rate of RTS at pre-injury levels being 65.2%, figures which are comparable to short-term follow-up periods ranging from 4.5 to 36 months in other studies which had rates of RTS ranging from 37 to 100% [22,23,24,25,26,27,28]. These short-term follow-up studies also had a rate of RTS at a pre-injury level ranging from 25 to 100%. Despite a high rate of RTS at a minimum 5-year follow-up, more relevant variables to be considered are the rate of continuation of a sport and the level of involvement at latest follow-up. The rate of RTS figures may be misleading, as athletes may participate in their respective sports post-operatively, but the level of involvement at latest follow-up fails to be mentioned in the majority of studies, a variable which is very valuable when counseling athletes regarding long-term outcomes. The study by Bauer et al. [15] was the only one that measured the level of involvement in sports at latest follow-up using the sports activity level score established by Valderrabano et al. [21] using the following scale: grade 0 (no sports activity); grade 1 (moderate level leisurely sports activity, <1 h/wk); grade 2 (normal level leisurely sports activity, 1–5 h/wk); grade 3 (high level leisurely sports activity, >5 h/wk); and grade 4 (professional/elite level of sports activity). Using this scale, Bauer et al. [15] found that though there was a high rate of RTS at 84.8%, the Valderrabano activity level at latest follow-up had only a mean of 1.6 ± 1.1 (1–5 h of sports activity per week), a relatively low level of activity. This was therefore determined to favor the high rate of RTS even though the level of involvement was low. Activity level scales such as this one established by Valderrabano et al. [21] are therefore vital for future studies to include when evaluating the rate of RTS in athletes following primary shoulder arthroscopy.

The overall rate of recurrent instability was 17.3%, with redislocation specifically occurring in 13.7%, and the overall rate of revision surgery was 11.1%. Rates of recurrent instability, redislocation, and revision surgery were slightly less than those reported in another systematic review, analyzing outcomes of the arthroscopic Bankart repair at a 10-year follow-up in cohorts mixed with athletes and non-athletes. Of 822 total shoulders, the overall rate of recurrent instability in that systematic review was 31.2%, with 16% of patients having recurrent dislocations, and an overall revision rate of 17% [29]. High rates of recurrent shoulder instability have been attributed to factors such as a glenoid bone loss of >15%, in which case the open Latarjet procedure is indicated. This places emphasis on appropriate patient selection and the weighting of pros and cons of arthroscopic interventions such as the Bankart repair and the open Latarjet repair, because even though the latter decreases the rate of recurrent instability, it also results in a distortion of normal anatomy which significantly restricts the range of motion, an outcome very important for athletes to consider and for surgeons to address [30,31]. Only two studies [17,19] in our systematic review mentioned significant glenoid bone loss as an exclusion criteria, and these two studies had a combined recurrence rate of 15.6%, whereas the three other studies had a combined recurrence rate of 18.8%. Though there was a slight difference in recurrence rates between these studies, statistical significance was not determined.

This systematic review assessed outcomes in athletes at a minimum 5-year follow-up. Therefore, studies which had a mean follow-up of five or more years were not included if the minimum value of the range of the follow-up period was less than 5 years. Other mid- to long-term studies should, however, be considered. Wilbur et al. [32] compared outcomes of the nonoperative and operative management of anterior shoulder instability in overhead (OHA) and non-overhead (NOHA) athletes (167 patients) at a mean follow-up of 11.9 (0.5–24.8) years. OHAs were more likely to present with subluxations, whereas NOHAs were more likely to present with dislocations. After initial nonoperative management and after surgery (arthroscopic, open with soft tissue repair, or open with bony augmentation), there were no differences in the rates of recurrent instability and revision surgery, RTS, RTS at preinjury level, time to RTS, and the WOSI score in OHAs and NOHAs. Additionally, we found comparable rates or values of all these variables across the studies included in our present systematic review. In another study, Till et al. [33] utilized unsupervised machine learning to define which patients undergoing surgery for anterior shoulder instability achieved an optimal outcome, and what were predictors of that outcome. At a mean follow-up of 11 years, 64% of patients achieved an optimal outcome, of which 41% achieved a perfect outcome. Patients with a suboptimal outcome had significantly higher rates of recurrent pain and instability after their initial surgery, and at final follow-up they had significantly higher rates of symptomatic osteoarthritis in addition to a lower degree of forward elevation. Furthermore, the artificial intelligence determined that the time from initial instability and the habitual/voluntary instability were negative predictors of optimal outcomes, whereas subluxations rather than dislocations before surgery were positive predictors. These findings can therefore be used when consulting athletic patients regarding potential long-term outcomes based on their specific presentation.

One study in this review had a low risk of bias [16], three had a moderate risk of bias [15,17,18], and another had a high risk of bias [19]. The study by Hurley et al. [16] had a low risk of bias as all it lacked was that data were not collected prospectively. It was, however, the only study to have an LOE of III. In addition to not collecting data and calculating study size prospectively and having a loss to follow-up of greater than 5%, Privitera et al.’s study [19] was the only one that clearly did not include consecutive patients. Therefore, they had a high risk of bias, and results from their study should be cautiously interpreted in the setting of this review. A loss to follow-up of greater than 5% was present in three of the included studies; however, these three studies had the longest follow-up periods of greater than 10 years, so it is a natural occurrence that a greater proportion of patients may have been lost. To strengthen their methodologies, future studies should consider prospectively collecting data and calculating their study size, in addition to clearly identifying an unbiased assessment of study points. Additionally, studies in athletes should specify sport type and competition level, and should report the time to RTS in addition to sports level at follow-up.

This systematic review has several strengths. First, it captures outcomes and rates of RTS, recurrent instability, and revision surgery in athletes at a minimum 5-year follow-up after primary shoulder arthroscopy. Second, this study reports on PROMs such as the Rowe and WOSI scores to contextualize outcomes from the patients’ perspectives. Third, this study provides detailed information from included studies on aspects such as factors associated with surgical and radiographic outcome. Fourth, surgical treatment was the same across all five studies (arthroscopic Bankart repair), which allowed for more comparable outcomes.

This systematic review also has limitations that must be addressed. First, all studies had retrospective study designs with a LOE of only III or IV. Second, no study mentioned any PROMs preoperatively, nor were there any clinical psychometric measures used which did not allow us to determine whether postoperative PROMs demonstrated significant improvement. Fourth, RTS is an ambiguous self-reported term which may hold different meanings in different patients. Fifth, some patients may not be interested in their respective sports at a minimum of 5 years after their surgical procedure, which may skew the rate of RTS figure. Sixth, athlete level was not mentioned in all studies, which may impact the rate of RTS and recurrent instability. Seventh, there were a limited number of studies present in this review, particularly due to our specific study topic of arthroscopic outcomes in athletes at a minimum 5-year follow-up, which therefore limits the strength of the conclusions derived from this review.

## 5. Conclusions

Athletes who underwent primary shoulder arthroscopy demonstrated favorable outcomes and a high rate of RTS at a minimum follow-up of 5 years. However, rates of recurrent instability, redislocation, and revision surgery occurred in less-than-favorable numbers, which emphasizes the importance of proper patient selection when considering candidates for arthroscopic versus open repairs. Further studies which compare long-term outcomes of arthroscopic and open techniques in athletes specifically are necessary to derive further conclusions.

## Figures and Tables

**Table 1:** Search strategies in PubMed, Embase, and Scopus.

PubMed	((shoulder arthroscop*) AND ((athlete) OR (sport))) AND ((((((((outcome) OR (survivorship)) OR (5-year)) OR (10-year)) OR (mid-term)) OR (long-term)) OR (five year)) OR (ten year))
Embase	(‘shoulder’/exp OR shoulder) AND arthroscop* AND (‘athlete’/exp OR athlete OR ‘sport’/exp OR sport) AND (‘outcome’/exp OR outcome OR ‘survivorship’/exp OR survivorship OR ‘5 year’ OR ‘10 year’ OR ‘mid term’ OR ‘long term’ OR ‘five year’ OR (five AND year) OR ‘ten year’ OR (ten AND year))
Scopus	TITLE-ABS-KEY ((shoulder AND arthroscop*) AND (athlete OR sport) AND (outcome OR survivorship OR 5-year OR 10-year OR mid-term OR long-term OR ( five AND year) OR (ten AND year)))

**Table 2:** Methodological quality and risk of bias in included studies.

Author	Clearly Stated Aim	Inclusion of Consecutive Patients	Prospective Data Collection	Endpoints Appropriate to Study Aim	Unbiased Assessment of Study Endpoint	Follow-Up Period Appropriate to Study Aim	Loss to Follow-Up Less than 5%	Prospective Calculation of Study Size	Total Score
Bauer et al. (2023) [15]	2	1	2	2	1	2	0	0	10/16
Hurley et al. (2022) [6]	2	2	0	2	1	2	2	2	13/16
Alentorn-Geli et al. (2016) [17]	2	1	0	2	1	2	2	0	10/16
Owens et al. (2009) [18]	2	1	2	2	1	2	0	0	10/16
Privitera et al. (2011) [19]	2	0	0	2	2	2	0	0	8/16

**Table 3:** Patient demographics and level of sports involvement.

Author	LOE	Study Type	Study Period	Number of Shoulders	Mean Follow-Up Time	Average Age at Surgery	Sport Type and Competition Level
Bauer et al. (2023) [15]	IV	Case Series	2001–2008	46	14.0 ± 1.8 years (11–18 years)	21.6 ± 4.5 years (18–30 years)	Not Reported
Hurley et al. (2022) [16]	III	Retrospective Cohort Study	2012–2015	144	75.7 ± 13.6 months (60–96)	26.9 ± 8.1	9 professional athletes, 95 competitive athletes, and 40 recreational athletes; 102 of the 144 total patients were collision athletes
Alentorn-Geli et al. (2016) [17]	IV	Cross Sectional Case Series	2002–2009	57	8 years ^1^ (5–10 years)	22 years ^1^ (16–28 years)	All competitive soccer players (Tegner score of 9)
Owens et al. (2009) [18]	IV	Case Series	March 1992–November 1998	40	11.7 years (9.1–13.9 years)	20.3 years (17–23 years)	1 collegiate soccer player, 39 unspecified athletes in the military
Privitera et al. (2011) [19]	IV	Case Series	1992–1999	20	13.5 years (10.75–17.5 years)	25 years (15–56 years) ^2^	All recreational

^1^ Median reported value, ^2^ average age at initial injury.

**Table 4:** Indications, intraoperative findings, PROMs, and postoperative radiographic findings.

Author	Surgical Indication	Surgical Intervention	Intraoperative Findings	PROMs at Latest Follow-Up	Radiographic Findings at Latest Follow-Up	Patient Satisfaction
Bauer et al. (2023) [15]	Primary traumatic anterior-inferior shoulder instability	Bankart repair	Complete labral lesion (16), SLAP lesion (4), Hill–Sachs lesion (27)	Constant score: 96.8 ± 5.1 Rowe score: 83.4 ± 24.4 WOSI: 90.7 ± 12.4	Anchors still visible: 12 Bone marrow edema: 11 Joint effusion: 14 Cysts: 9 Osteoarthritis: 9 ^2^	42 satisfied 4 dissatisfied (all 4 had redislocation and 1 also had developed osteoarthritis)
Hurley et al. (2022) [16]	Traumatic anterior shoulder instability	Bankart repair	Off-track Hill–Sachs lesion (11)	VAS: 2.1 ± 2 SIRSI: 63.7 ± 25.7 SSV: 85.8 ± 14.4	Glenoid bone loss: 1.9% ± 4.1%	Satisfaction on scale of 1–5: 1 (1 patient) 2 (6 patients) 3 (18 patients) 4 (43 patients) 5 (76 patients) 121/144 would undergo surgery again
Alentorn-Geli et al. (2016) [17]	Anterior glenohumeral instability	Bankart repair	Bankart lesions (46), anterior labroligamentous periosteal sleeve avulsion (11), Hill–Sachs lesions (all), type I SLAP lesion (5), type II SLAP lesion (3), Buford complex (2)	Rowe: 80 (25–100) ^1^ QuickDASH: 2.3 (0–12.5) ^1^ QuickDASH sports score: 0 (0–18.8) ^1^	Not Reported	Not Reported
Owens et al. (2009) [18]	First-time traumatic anterior glenohumeral dislocation	Bankart repair	Not Reported	SANE: 91.7 (40–100) WOSI: 371.7/82.3% (9–1875) ROWE subjective: 25.3 (0–30) SST: 11.1 (6–12) ASES: 90.9 (31.7–100) SF-36 Physical Component: 94.4 (25–100)	Not Reported	Would have surgery again: mean of 9.1 (1–10-point scale)
Privitera et al. (2011) [19]	Anterior shoulder instability	Bankart repair; concomitant radiofrequency capsulorrhaphy due to capsular laxity (2)	Bankart lesion (18), glenoid chondromalacia (5)	WOSI: 80% (physical: 77%, sports/rec: 80%, lifestyle: 87%, emotions: 77%) DASH Main: 7.3 (0–39.2) DASH Work: 7.64 (0–44) DASH sports/arts: 14.17 (0–69)	Absent arthrosis: 4/20 Mild arthrosis: 8/20 Moderate arthrosis: 5/20 Severe arthrosis: 3/20 Drill holes used to implant tacks invisible in 8 shoulders, hardly visible in 3 shoulders, and clearly visible in 9 shoulders (less than 3 mm in 5 shoulders and greater than 3 mm in 4 shoulders) Absent Hill–Sachs lesions: 9/20 Small Hill–Sachs lesions: 10/20 Moderate Hill–Sachs lesions: 1/20	Not Reported

^1^ Median reported value, ^2^ MRI could be completed in only 32 of the 46 patients in this study.

**Table 5:** Return to sports and rate of recurrent instability.

Author	Number of Patients Returning to Sports	Number of Patients Returning to Sports at Pre-Injury Level	Time to RTS	Sports Level at Follow-Up	Rate of Recurrent Instability	Time from Surgery to Recurrent Instability
Bauer et al. (2023) [15]	39/46 (84.8%)	38/46 (82.6%)	6.3 ± 3.0 months	Valderrabano Sports Level: 1.6 ± 1.1 (1–5 h of sports activity per week)	Redislocations: 10 Revision surgery: 6	31.8 ± 32.5 months (7–108 months) to first redislocation
Hurley et al. (2022) [16]	116/144 (80.6%)	92/144 (63.9%)	6.2 ± 2.7 months	Not Reported	Redislocations: 15 Subluxations: 3 Revision surgery: 15	Not Reported
Alentorn-Geli et al. (2016) [17]	49/57 (86%) 8 who did not RTS quit because of non-shoulder reasons	36/57 (63.2%)	4 months (3–5 months) ^1^	Not Reported	Redislocations: 6 Revision surgery: 4 All redislocations of traumatic origin	Not Reported
Owens et al. (2009) [18]	39/39 (100%)	Percentage of pre-injury shoulder function: 93.3% (40–105%)	Not Reported	Tegner: 6.5 (3–10) APFT: 287.45 Number of push-ups in two minutes: 72.8 (20–100)	Redislocations: 6 Subluxations: 9 Revision surgery: 6	37 months to redislocation, 22 months to subluxation
Privitera et al. (2011) [19]	14/20 (70%) 4 quit because of shoulder problems; 2 not interested in sports without shoulder problems	8/20 (40%) (6/20 at limited level)	Not Reported	Not Reported	Redislocations: 5 Subluxations: 1 Instability: 1 Revision surgery: 3 6/7 instances due to trauma	4.2 years (0.25–14.7 years)

^1^ Median reported value.

## Data Availability

Not applicable.
